# Fractured Minds: A chronicle of neurotrauma in the 18^th^ century

**DOI:** 10.25122/jml-2023-1028

**Published:** 2023-09

**Authors:** Stefana-Andrada Dobran, Livia Livint Popa, Dafin Muresanu

**Affiliations:** 1 RoNeuro Institute for Neurological Research and Diagnostic, Cluj-Napoca, Romania; 2 Sociology Department, Babes-Bolyai University, Cluj-Napoca, Romania; 3 Department of Neuroscience, Iuliu Hatieganu University of Medicine and Pharmacy, Cluj-Napoca, Romania

## INTRODUCTION

The exploration of neurotrauma over the course of history has significantly influenced modern science, providing valuable perspectives on the cultures and civilizations throughout time. Significant strides in understanding neurotrauma, particularly concussive disorders, were made during the 18^th^ century, unveiling its origins and offering a better understanding of the mechanisms of cerebral pulsation, skull vibration, intracranial pressure increase, and management of concussion. Moreover, the century brought forward striking progress in neurotrauma surgery. While the Arabic physician Rhazes introduced the concept of concussion in the 9^th^ century, it was in the 16^th^ century that the affection gained more comprehensive attention. As the Age of Enlightenment represented a cornerstone in understanding the pathophysiological mechanisms, leading the path towards the scientific method, the study of concussion in the 18^th^ century marked a pivotal moment in the history of neurotrauma and neurosurgery [1, 2].

## THE MEDICAL HISTORY OF NEUROTRAUMA IN THE 18^TH^ CENTURY

Humphrey Ridley (1653–1708) was a pioneer in recognizing the vascular origin of cerebral pulsation, a breakthrough that laid the foundation for understanding neurological processes in neurotrauma. Dupuytren (1777–1839) later coined the term ‘concussion’, further refining our grasp of traumatic brain injuries (TBIs) [2, 3]. He not only labeled the phenomenon but also described the pathophysiological mechanism of the skull vibration resulting from an injury that leads to a brief loss of consciousness. This exploration marked a crucial step in unraveling the intricacies of neurotrauma. Additionally, the 18^th^ century also witnessed the first strides in attributing the increase of intracranial pressure to epidural hematoma, offering early insights into the physiological consequences of brain injuries [3].

At that time, the management of concussions was a subject of considerable controversy, with opinions differing on the appropriate course of action. Percival Pott was one of the pioneers who supported indications for surgery based on the neurological condition. In France, Francois Quesnay (1694–1774), the physician of Louis XV, joined Pott in advocating for prophylactic trepanning in cases with localized signs. In contrast, others advocated for more conservative treatments, contending that compression can be temporary and not necessarily invasive [3].

Another figure of the time, John Hunter (1728–1793), offered detailed observations on compression, concussions, and brain lacerations, as well as on the correlation of awareness with pulse, respiration, and pupillary response. Hunter’s approach to trepanning was limited to cases with depressed fractures and open dura mater, excluding its use in isolated concussions [3]. The 18^th^ century brought a paradigm shift in the understanding of post-injury alteration of the mental state. Unlike earlier assumptions linking it exclusively to skull damage, it was then seen as being related to brain pressure [4]. This shift in perspective represented a significant advancement in the conceptualization of neurotrauma.

Certain scientific advancements, such as the creation of the microscope in the 17^th^ century and the advent of the Age of Enlightenment in the 18^th^ century, propelled medical minds to a pathophysiological understanding of concussion, achieved through animal experimentation and case studies. This changed the presumption of concussion as a transient phenomenon and offered new insights into the clinical neurological changes, in contrast to the previous approach based exclusively on clinical observation [5]. Jean-Louis Petit played a significant role in shaping the idea of concussion as a symptom, attributing the immediate loss of consciousness to the concussion. Another distinguished scientist, John Bell, introduced clinical symptoms as a diagnostic tool for distinguishing among different types of TBI: concussion, compression, and inflammation [5].

However, progress has led to functional theories replacing structural theories on TBI. Additional theories emerged, including the hypothesis of circulatory failure resulting in venous congestion, put forward by Littre (later disapproved), the hypothesis of ‘cerebral anemia’ (also known as acute compressive anemia), the theory of molecular vibration proposed by Baudens, and the hypothesis of nerve cell shock introduced by Petit. The progress has led to two different schools of thought on TBI, structural versus functional, a debate that continues to the present day [5].

## NEUROSURGERY IN THE 18^TH^ CENTURY: DISSECTING THE PRACTICE

The 18^th^ century has seen remarkable progress in trauma neurosurgery compared to the few changes that have taken place in the field since ancient times. Skull injuries presented a particular interest, whereas post-trauma symptoms were mostly attributed to injuries of the bones and meninges. Surgeons of the time included Henri-François Le Dran (1685–1770) in France, Percival Pott (1714–1788) and John Abernethy (1764–1831) in England, Sylvester O’Halloran (1728–1807) and William Dease (1750–1798) in Ireland, and James Hill (1703–1776) in Scotland [2]. Their collective efforts have laid the groundwork for the evolving landscape of neurosurgical interventions.

The specialists from the Académie Royale de Chirurgie in Paris pioneered the notion that post-traumatic head injury symptoms stem from the brain. Le Dran published the first of these findings in 1740, recognizing the similarity between concussion and intracranial extravasation symptoms, and the significance of cerebral tension and pulsation as indexes of cerebral health. In 1708, he implemented the first successful treatment of epidural hematoma, marking the first step towards reducing increased intracranial pressure [2].

Percival Pott, who was under the mentorship of the leading surgeon at St. Bartholomew’s hospital since the age of 15, gained invaluable experience by treating 43 head injuries and writing accounts of their case histories. Pott considered that head trauma symptoms originate in brain damage due to the brain being violently shaken or experiencing abnormal pressure, or a derangement of the medullary structure [2]. James Hill of Dumfries, the author of ‘Cases in Surgery’ (1772), described 18 cases of cranial trauma, demonstrating a great understanding of the management of severe intracranial damage and refining the practices of the time. Hill and Abernethy were the first to recognize the importance of cerebral lateralization [2].

In the 18^th^ century, alterations in consciousness were increasingly accepted as the reflection of brain injury. However, the explanations for the phenomenon were generally lacking and, where present, were related to the concept of the soul, a perspective that prevailed until the end of the century.

## THROUGHOUT THE CENTURIES: LESSONS FROM THE STUDY OF NEUROTRAUMA

Although neurotraumatology might not seem to be the most glamorous among the subdomains of neuroscience, its study has shaped humanity since the dawn of time. The passion for unraveling the mechanisms of injury and the dedication to finding solutions have driven physicians and other professionals to develop numerous tools and techniques that have become commonplace in today’s medical field. Understanding the complex effects of neurotrauma is no simple task, as it affects the individual at the physiological, psychological, and social levels. Consequently, understanding the history of neurotrauma provides a profound appreciation for the efforts of specialists who paved the way, fostering a sense of gratitude for the progress achieved through collective work.

## References

[1] Seymour B (2013). Defining Concussion and Mild Traumatic Brain Injury: a History of Confusion and Debate. https://soundideas.pugetsound.edu/soundneuroscience/vol1/iss1/9.

[2] Ganz JC (2013). Head injuries in the 18th century: the management of the damaged brain. Neurosurgery.

[3] Bertullo G (2015). History of Traumatic Brain Injury (TBI). AJBM.

[4] Granacher RP (2007). Traumatic Brain Injury: Methods for Clinical and Forensic Neuropsychiatric Assessment.

[5] McCrory PR, Berkovic SF (2001). Concussion: the history of clinical and pathophysiological concepts and misconceptions. Neurology.

